# Impact of Electrolyte Formulation on the Phase Behavior and Interphase Formation of Sb/Graphite Electrodes for K‐Ion Batteries

**DOI:** 10.1002/advs.76057

**Published:** 2026-06-11

**Authors:** Ezzoubair Bendadesse, Max Wacha, Zeynep Erdol, Yanan Sun, Carsten Prinz, Mirko Boin, Manuela Klaus, Tatiana Mishurova, Till Wolfram, Jürgen Janek, Philipp Adelhelm

**Affiliations:** ^1^ Institut für Chemie Humboldt Universität zu Berlin Berlin Germany; ^2^ Physikalisch‐Chemisches Institut Justus‐Liebig‐Universität Gießen Gießen Germany; ^3^ Joint Research group for operando battery analysis (CE‐GOBA) Helmholtz‐Zentrum Berlin für Materialien und Energie (HZB) Berlin Germany; ^4^ BAM Federal Institute For Materials Research and Testing Berlin Germany; ^5^ Dept. Microstructure & Residual Stress Analysis (CE‐AME) Helmholtz‐Zentrum Berlin für Materialien und Energie (HZB) Berlin Germany; ^6^ Wolfram Chemie GmbH Berlin Germany

**Keywords:** alloy anodes, operando analysis, potassium‐ion batteries, Sb‐graphite

## Abstract

High capacity alloy‐type materials are attractive anodes for potassium‐ion batteries yet their practical use is hampered by extreme volumetric expansion that causes mechanical instability and rapid capacity fading. Here we show that the electrolyte formulation decisively governs both the electrochemical performance and structural integrity of a high‐capacity Sb/graphite composite anode (70:30 wt.%). A localized high‐concentration electrolyte (LHCE) comprising KFSI, glyme solvents, and 1,1,2,2‐tetrafluoroethyl‐2,2,3,3‐tetrafluoropropyl ether as diluent delivers markedly improved durability, sustaining over 300 cycles with 400 mAh g^−1^ (1.5 mAh cm^−2^), whereas a conventional carbonate‐based electrolyte (CBE) exhibits rapid degradation. Operando Raman spectroscopy, operando energy‐dispersive X‐ray diffraction, operando electrochemical dilatometry, and ex situ XPS and TEM reveal that the benefit arises from a two‐dimensional electrolyte effect on both surface and bulk electrode behavior. The CBE promotes crystalline multiphase K–Sb alloying together with pronounced graphite participation and forms a thick, organic‐rich SEI, leading to large, poorly reversible swelling and mechanical damage. In contrast, the LHCE favors predominantly amorphous K_x_Sb formation, suppresses deep K^+^ intercalation into graphite, and forms a thin inorganic, KF‐rich interphase that mitigates internal strain. These insights link solvation, interphase chemistry, and chemo‐mechanics, guiding electrolyte design for stable alloy anodes.

## Graphical abstract

Electrolyte formulation is shown to govern both interphase chemistry and bulk phase evolution in Sb/graphite anodes for potassium‐ion batteries. A diglyme‐based localized high‐concentration electrolyte promotes a thin inorganic‐rich interphase, suppresses deep graphite potassiation, limits electrode swelling, and stabilizes amorphous K_x_Sb formation, enabling markedly improved cycling durability.

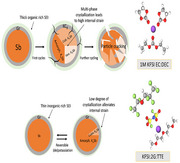

## 1 Introduction

The growing demand for sustainable and high‐performance energy storage systems has driven extensive research into alternative battery chemistries beyond lithium‐ion technologies. Among these, potassium‐ion batteries (PIBs) have emerged as a promising candidate due to potassium's natural abundance, low cost, and the ability to use aluminum current collectors for both electrodes, reducing overall cell cost and weight [1]. However, the relatively large ionic radius of K^+^ (1.38 Å vs. 0.76 Å for Li^+^) poses significant challenges for electrode materials, particularly with respect to diffusion kinetics, structural integrity, and electrode–electrolyte interfacial stability [2, 3, 4].

To overcome these issues, alloy‐type anodes, such as antimony (Sb), have garnered attention for their high theoretical capacity (660 mAh g^−^^1^ for K_3_Sb) and relatively low redox potential (0.35 V vs. K^+^/K) [5, 6, 7]. Nonetheless, they suffer from substantial volume changes (400%) during (de)potassiation, leading to particle pulverization, an unstable solid electrolyte interphase (SEI), and rapid capacity fade [8]. To address these challenges, various strategies have been explored, including nanoscaling Sb particles to accommodate volume changes [9, 10], developing high‐entropy alloys with enhanced structural stability [11, 12], and constructing composite electrodes that integrate alloying materials with carbonaceous matrices to buffer mechanical stress and improve electronic conductivity [13, 14, 15]. However, the effectiveness of such composites is closely tied to the electrolyte environment, which dictates both solid electrolyte interphases (SEI) composition and the extent of side reactions.

Recent developments in electrolyte engineering have led to the emergence of localized high‐concentration electrolytes (LHCEs), which offer a promising approach to stabilizing alloy‐type anodes. These electrolytes combine a high salt concentration with inert diluents that reduce viscosity and cost without disrupting the local coordination structure [16, 17, 18]. In LHCEs, the high local salt content reshapes the solvation environment around K^+^ by favoring the formation of contact ion pairs (CIPs) and ion aggregates (AGGs). Consequently, anions such as FSI^−^ become more involved in the primary solvation shell of the cation. This shift in solvation structure has a direct impact on the electrolyte's electronic properties, the increased participation of anions in the primary solvation shell lowers the LUMO of the solvated ionic complex and shifts its character toward the anion. This makes anions more susceptible to reduction at the electrode surface during initial cycling. As a result, the SEI that forms is predominantly composed of inorganic species like KF and K_2_SO_3_ [19], which are known to enhance chemical and mechanical stability. Compared to the organic‐rich SEIs formed in conventional carbonate‐based electrolytes—where solvent reduction is dominant—these inorganic layers tend to be more stable, ion‐conductive, and better at accommodating the volume changes associated with alloying reactions. While these SEI‐related benefits of LHCEs are well established, relatively little attention has been paid to how these electrolytes influence the electrochemical behavior of the bulk electrode, particularly in composite systems that include graphitic carbon. In such electrodes, the solvation structure of the electrolyte does not just affect interfacial reactions; it also governs how easily K^+^ ions desolvate and whether they intercalate into the graphitic domains. Depending on the electrolyte formulation, this intercalation can be either suppressed or promoted—affecting not only capacity but also the mechanical integrity of the electrode [20]. Understanding how LHCEs influence both surface chemistry and bulk ion dynamics is therefore crucial for designing stable and efficient alloy–carbon composite anodes.

In this study, we investigate the interplay between SEI composition and bulk structural effects in a composite electrode comprising 70 wt.% Sb and 30 wt.% graphitic carbon, prepared by high‐energy ball milling (HEBM). We evaluate its electrochemical behavior in a conventional electrolyte (1 M KFSI in EC:DEC) and three LHCEs formulated with monoglyme (1G), diglyme (2G), or a 1:1 molar mixture of both, each diluted with TTE (1:2:1 molar ratio of salt:solvent:diluent). The selected LHCE formulations were literature‐guided and chosen to preserve a comparable salt:solvent:diluent ratio while varying the coordinating glyme environment. KFSI was selected because of its high solubility and widespread use in ether‐based potassium electrolytes, while the fluorinated ether diluent was used to lower viscosity and improve practical transport properties without substantially disrupting the local solvation structure. Conventional KFSI/glyme electrolytes were also considered, but were not used for direct comparison because graphite storage in such electrolytes depends strongly on salt concentration and can involve concentration‐dependent co‐intercalation effects, complicating interpretation of the Sb/Gr composite response.

The electrolytes and their composition are reported in Table S1 of the Supporting Information file. All LHCEs show improved capacity retention compared to the conventional electrolyte, with diglyme‐containing LHCEs (LHCE‐G2 & LHCE‐G1/G2) outperforming the monoglyme‐based LHCE‐G1. X‐ray photoelectron spectroscopy (XPS) revealed that the SEI formed in the LHCEs is richer in inorganic, salt‐derived species compared to that generated in the carbonate‐based electrolyte. In situ Raman spectroscopy further indicates that K^+^ intercalation into the graphitic component of the composite is largely suppressed in LHCE‐G2, in contrast to the pronounced intercalation observed in the carbonate electrolyte. Operando Energy‐Dispersive X‐ray Diffraction (ED‐XRD) corroborates these findings, showing distinct reflections of crystalline K─Sb phases in the carbonate electrolyte that are absent in LHCE‐G2. The combination of multiphase potassiation in the Sb/Gr composite and simultaneous K^+^ intercalation into graphite in the carbonate electrolyte induces severe internal mechanical strain, consistent with the significantly higher volumetric expansion observed by operando dilatometry.

These findings suggest that the improved performance of LHCEs arises not only from their interfacial passivation but also from a bulk‐suppressive effect on the graphitic component, which reduces internal strain in the composite and enhances cycling stability.

## 2 Results and Discussion

**FIGURE 1:**
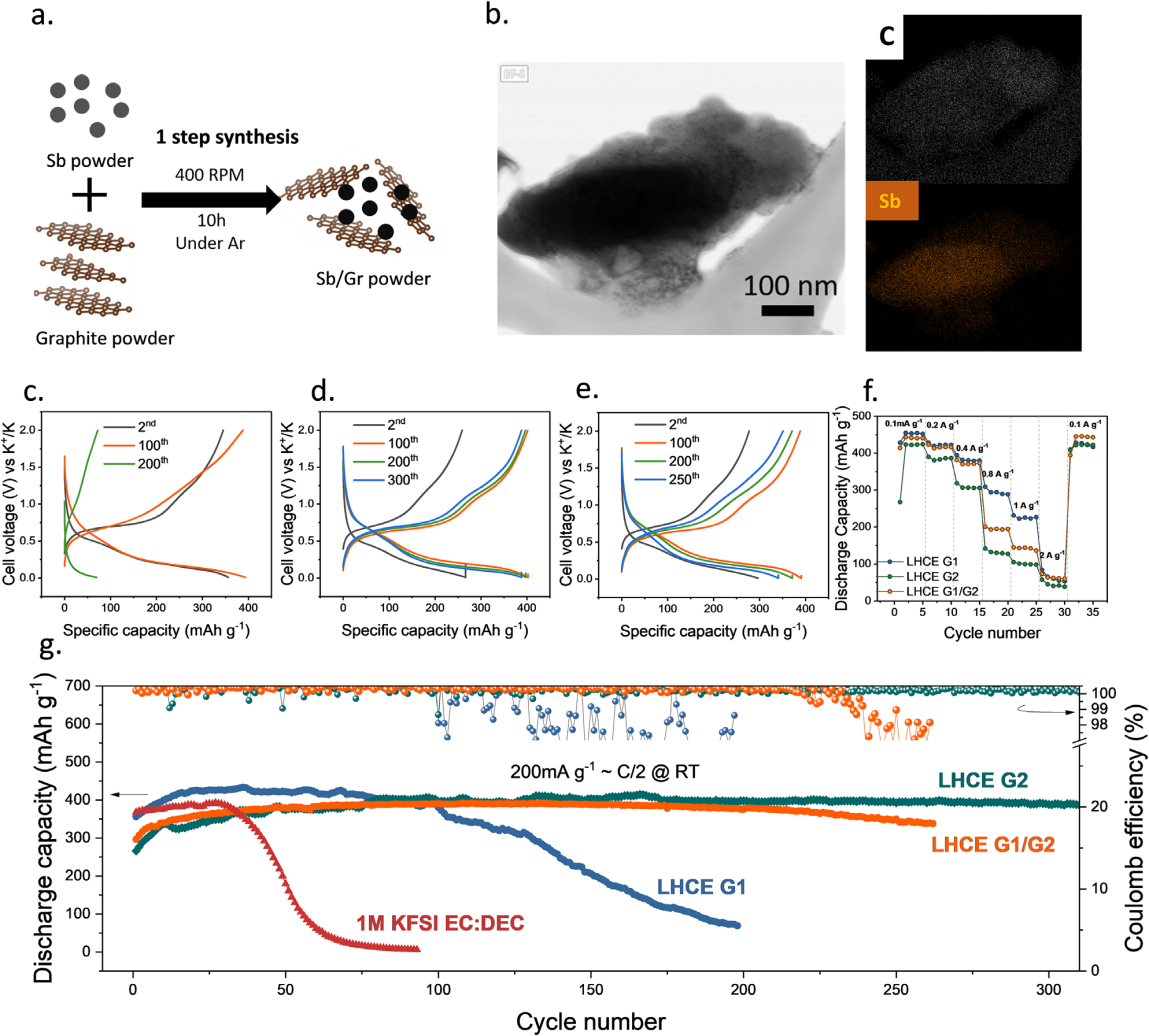
(a) Synthesis of the Sb/Gr composite by high‐energy ball milling (HEBM). (b) TEM image of an Sb/Gr particle with corresponding EDX elemental maps of C and Sb. Voltage profiles of the second, 100th, 200th, and 300th cycles of Sb/Gr in (c) LHCE‐G1, (d) LHCE‐G2, and (e) LHCE‐G1/G2 using two‐electrode measurements with K metal as counter electrode. (f) Rate‐capability performance of Sb/Gr electrodes in the different LHCE formulations. (g) Capacity‐retention behavior of Sb/Gr electrodes in all tested electrolytes, cycled at 200 mA g^−^^1^ (≈C/2 rate).

The Sb/Gr composite was synthesized through high‐energy ball milling, combining commercial antimony and graphite powders in a 70:30 weight ratio (Figure 1a). Further details of the synthesis procedure are provided in the Supplementary Information. The mechanical synthesis procedure yields a core‐shell‐like structure where Sb particles are embedded in a carbon shell, as seen in TEM images (Figure 1b). This structure helps accommodate the extreme volumetric expansion of Sb upon (de)potassiation and preserves the structural integrity of the active material particles. Additionally, thermogravimetric analysis (TGA) under an O_2_ atmosphere was performed to estimate the carbon content of the composite (Figure S1). A small mass increase of about 3% is first observed at around 390°C, which can be attributed to partial oxidation of Sb to Sb_2_O_3_. Upon further heating, two partially overlapping mass‐loss steps appear between about 410°C and 450°C, giving a total mass loss of ∼33%. This behavior likely reflects oxidation of carbon domains with different structural order, with more defect‐rich or amorphous carbon oxidizing at lower temperature and more ordered graphitic domains at higher temperature [21]; the value is in reasonable agreement with the nominal graphite content of 30 wt.%. At higher temperatures, a further mass‐loss step is observed near 500°C, corresponding to an additional ∼8% decrease, which is attributed to sublimation of antimony oxide, as previously reported in the literature [22].

Assuming the complete potassiation of both Sb (K_3_Sb) and graphite (KC_8_), the theoretical capacity is expected to reach 545 mAh g^−1^. Although the two components operate at distinct potentials (≈0.1 V for graphite and ≈0.35 V for Sb vs. K^+^/K), the later stages of K–Sb alloying occur at lower voltages that can overlap with the redox potential range of graphite, making the distinction between the participation of each component in the overall capacity challenging. Even assuming complete KC_8_ formation, the 30 wt.% graphite fraction could contribute at most ∼84 mAh g^−^^1^ to the overall composite capacity; the actual value is likely lower because Raman spectroscopy indicates partial amorphization of the graphitic component during HEBM (Figure S2).

To evaluate the electrochemical performance of the composite electrode, half‐cell measurements were carried out using three localized high‐concentration electrolytes: LHCE‐G1, LHCE‐G2, and LHCE‐G1/G2. For comparison, a fourth electrolyte based on a conventional formulation using a mix of carbonates as solvents: 1 M KFSI in EC:DEC (1:1 v/v), was also included, hereafter referred to as CBE. An inherent feature observed in all electrolyte systems is the irreversible capacity loss during the first potassiation, reflected in the low initial Coulomb efficiency (ICE) of about 65%. This behavior can be attributed to partial irreversible potassiation of Sb particles combined with SEI formation. As illustrated in Figure S3a, the first potassiation reveals two quasi‐plateau regions at 0.5 and 0.25 V versus K^+^/K for the CBE and LHCE‐G1, whereas LHCEs containing G2 display the plateaus at 0.25 and 0.1 V versus K^+^/K, accompanied by higher polarization induced by the more viscous diglyme solvent. During charging, a minor slope inflection at 0.28 V versus K^+^/K is visible in both the CBE and LHCE‐G1 but absent in the G2‐based LHCEs, while the potassiation (0.7 V vs. K^+^/K) and depotassiation (0.65 V vs. K^+^/K) processes remain comparable across all electrolytes, with G2‐containing systems exhibiting slightly increased hysteresis. CV profiles of the Sb/Gr electrode cycled in the selected electrolytes are shown in Figure S3b. During the cathodic scan, three reduction peaks are observed at approximately 0.65, 0.25, and 0.23 V versus K^+^/K, consistent with a multistep potassiation process involving K─Sb alloying together with possible K^+^ intercalation into the graphitic component. In LHCE‐G2, the last reduction process occurs with noticeably higher polarization, suggesting that the final potassiation step, commonly associated with K_3_Sb formation, is kinetically hindered in this electrolyte. During the anodic scan, three oxidation peaks appear at approximately 0.28, 0.6, and 1.2 V versus K^+^/K, indicating the overall reversibility of the alloying reaction. Notably, the low‐potential oxidation peak at 0.28 V is weaker and more polarized in LHCE‐G2, further supporting less complete formation of K_3_Sb under these conditions. Because deep graphite potassiation occurs in the same low‐potential region as the later stages of Sb alloying, the galvanostatic profiles of the Sb/Gr composite cannot be rigorously deconvoluted into independent Sb and graphite capacity contributions. Graphite participation will be determined qualitatively later in the manuscript.

The galvanostatic charge–discharge profiles of the half cells at selected cycles (Figures 1c–e) highlight the distinct long‐term cycling behavior of the various LHCE formulations. All three electrolytes exhibit stable potassiation/depotassiation plateaus and sloping regions up to at least the 100th cycle, corresponding to the reversible formation of K–Sb phases [23]. Upon extended cycling, a clear trend emerges in which the G2‐containing electrolytes exhibit enhanced stability, characterized by minimal capacity fade and polarization decrease, with LHCE G2 demonstrating the best overall performance. In contrast, LHCE G1 displays the most pronounced capacity degradation and increasing overpotential over time (Figure 1c).

Interestingly, a gradual capacity recovery is observed within the first 100 cycles in all LHCE systems, with improvements of approximately 14%, 51%, and 44% for LHCE G1, LHCE G2, and LHCE G1/G2, respectively. This trend is attributed to a progressive reduction in overpotential associated with the potassium metal counter electrode as well as an improvement in electrode wetting over time due to the higher viscosity of LHCEs. Supporting evidence from symmetrical potassium metal plating/stripping cells (Figure S4) shows a steady decline in overpotential by ∼150 mV after 100 h of cycling, suggesting improved potassium metal interface stability.

Figure 1f presents the rate performance of the different electrolytes. LHCE‐G1 exhibits superior capacity retention at high current densities (200 mAh g^−1^ at 1 A g^−1^) compared to the G2‐containing electrolyte (100 mAh g^−1^ at 1 A g^−1^), likely due to the lower viscosity of monoglyme, which facilitates faster ionic transport [24, 25]. In contrast, the higher viscosity of diglyme in LHCE‐G2 may hinder ion diffusion, leading to reduced performance at elevated rates. The mixed‐solvent system, LHCE‐G1/G2, provides a balanced compromise, combining improved ionic conductivity with moderate viscosity, resulting in satisfactory rate capability (144 mAh g^−1^ at 1 A g^−1^). Figure 1g highlights the superior long‐term cycling stability of the G2‐containing systems. LHCE‐G2 maintains a capacity of approximately 400 mAh g^−^^1^ after 300 cycles, while the mixed‐solvent LHCE‐G1/G2 retains around 350 mAh g^−^^1^ after 250 cycles. In contrast, LHCE‐G1 exhibits a gradual capacity decline beginning after the 100th cycle, and the conventional electrolyte performs significantly worse, showing an early capacity drop with a pronounced knee‐point before 50 cycles. These enhanced electrochemical performances motivated a deeper investigation into the underlying mechanisms, which are explored in the following sections through a combination of complementary characterization techniques.

### 2.1 Solvation Structure Probed by Raman Spectroscopy

**FIGURE 2:**
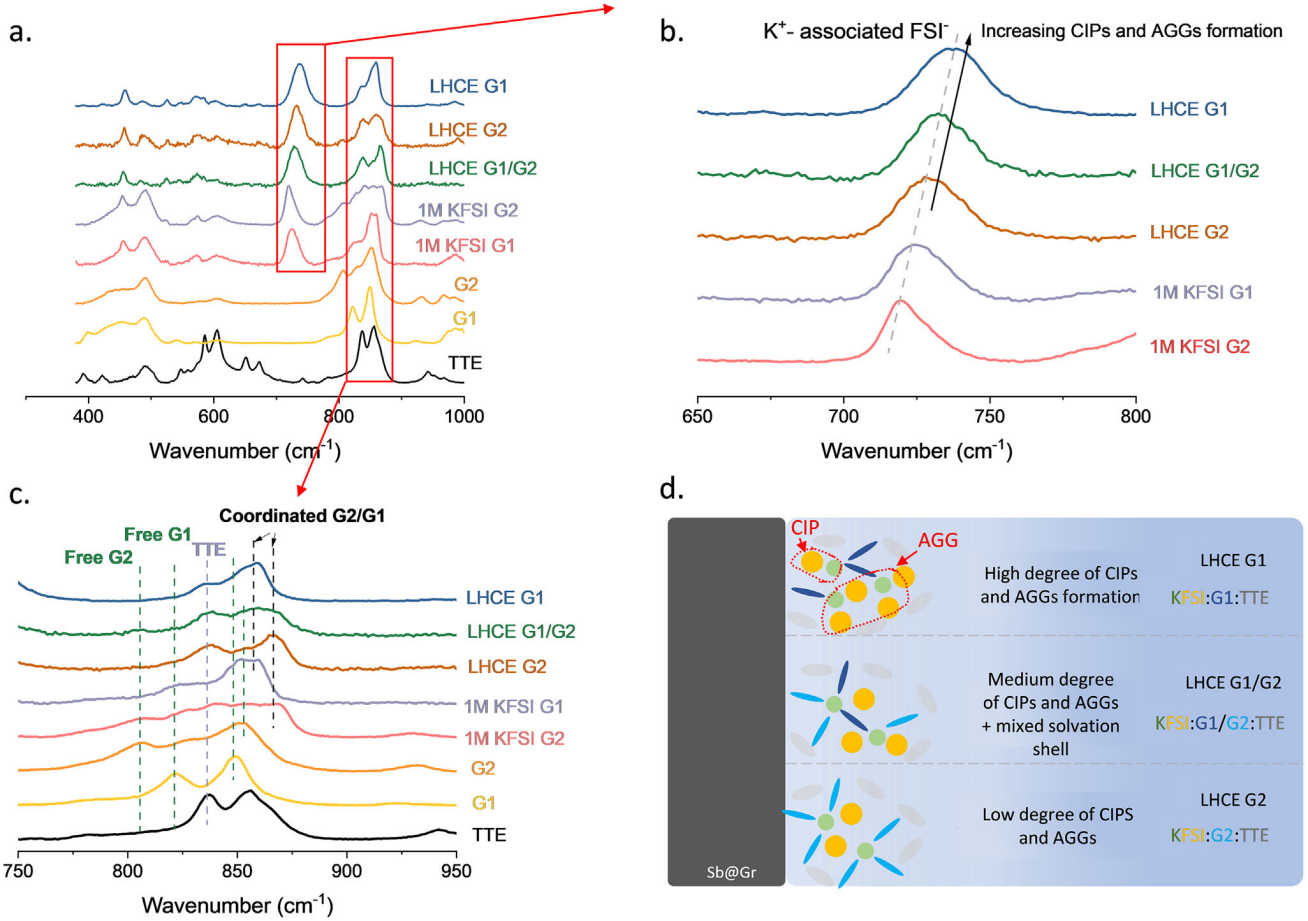
Raman spectroscopy analysis of the electrolytes. (a) General spectra of the LHCEs compared with reference electrolytes at conventional concentrations and the pure solvent. Zoomed‐in regions show (b) the FSI^−^‐related absorbance band and (c) solvent‐related absorbance bands. (d) Schematic representation of the solvation shell structure in the different LHCE formulations.

To investigate the solvation structure of the various LHCE formulations, Raman spectroscopy was conducted. The results are presented in Figure 2a. The analysis focuses on two key spectral regions: the vibrational modes of the FSI^−^ anion in the 650–800 cm^−^^1^ range, and the characteristic solvent bands between 800 and 900 cm^−^^1^. To accurately assign the solvent‐related bands, additional Raman measurements were performed on conventional electrolytes (1 M KFSI in G1 or G2) as well as on the pure solvents. In the first spectral region (Figure 2b), the peak corresponds to the S–N stretching vibration of the FSI^−^ anion [26]. A systematic blueshift of this peak is observed when moving from G2, to the mixed G1/G2 system, and finally to G1 as the sole solvent. This trend indicates a progressive increase in CIP and AGGs formation, reflecting a greater incorporation of FSI^−^ anions into the primary solvation shell of K^+^ in the presence of G1 [27, 28, 29]. The same behavior is also observed in the conventional electrolytes.

In the second spectral region (Figure 2c), associated with solvent vibrational modes, comparison with the pure solvents and conventional electrolytes allows free and solvating molecules to be distinguished within the overlapping peaks. Here, the coordinated solvent bands show a blueshift from G1‐containing electrolytes toward the G2‐based ones. This indicates that G2 molecules are more strongly incorporated into the solvation shell than G1, in line with the opposite trend found in the anionic region. In the mixed G1/G2 LHCE, a broader band spanning both solvent modes suggests a shared solvation shell, consistent with an intermediate degree of CIP and AGG formation. By contrast, the TTE band appears distinctly in the LHCE spectra but without any shift, confirming its lack of involvement in the solvation shell. A schematic of the ionic clusters in the LHCEs is shown in Figure 2d.

### 2.2 Electrolyte‐Dependent Stabilization of Sb/Gr Electrodes

**FIGURE 3:**
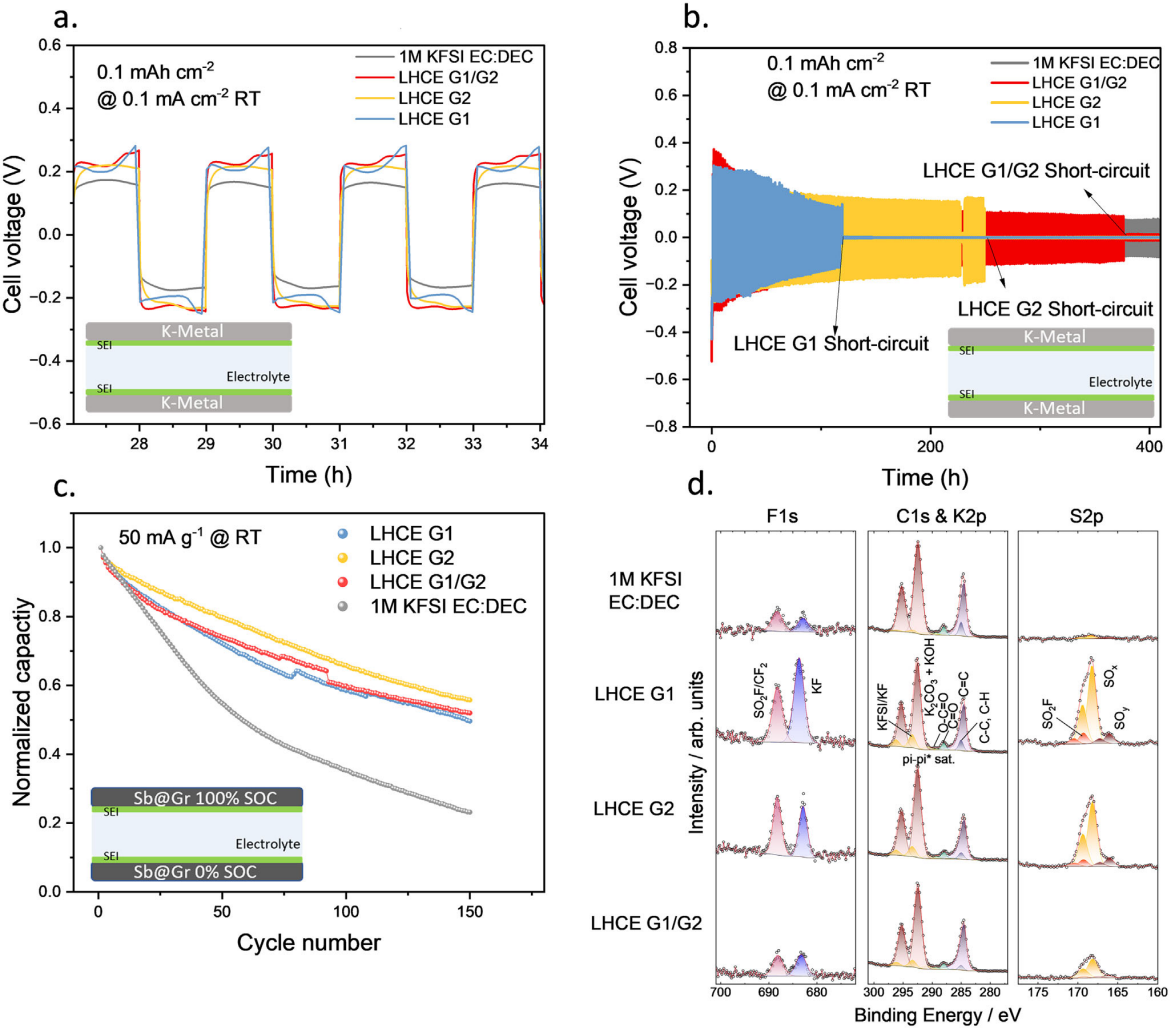
Symmetric cell studies of K|K and Sb/Gr|Sb/Gr systems. (a) K|K plating–stripping experiment in the selected electrolytes at a current density of 0.1 mA cm^−^^2^; (b) zoomed‐out view showing internal shorting events of K|K cells; (c) Capacity retention of Sb/Gr|Sb/Gr symmetric cells using the selected electrolytes at a current density of 50 mA g^−^^1^; (d) XPS spectra of the surface of Sb/Gr electrodes after their first discharge in the selected electrolytes showing the F1s, C1s, K2p, and S2p regions.

Symmetric cell tests were run in order to decouple the contributions of the electrolyte to the stability of the potassium metal and to the Sb/Gr electrode. Figure 3a,b shows the results for K|K plating/stripping cells assembled with either the CBE or the various LHCEs. In these configurations, we observe that all LHCEs exhibit higher polarization than the CBE, with the sequence LHCE‐G2> G1/G2> G1 (Figure 3a). Consistent with this, the CBE also provides superior cycling stability in symmetric K cells, whereas LHCE‐based cells undergo premature short circuiting, attributable to accelerated K‐metal dendrite growth (Figure 3b). These results confirm that LHCEs do not inherently suppress dendrite formation on potassium metal and that their beneficial effect cannot be ascribed to simple stabilization of the alkali metal surface. This observation is in line with plating/stripping experiments in Na‐ion cells containing LHCE for which excessive dendrite growth was observed due to a root‐growth mechanism [30].

By contrast, symmetric cells employing Sb/Gr composite electrodes at opposing states of charge show the opposite trend: here, capacity retention is significantly improved in the presence of LHCEs, with the same performance hierarchy (G2> G1/G2> G1) as observed in half‐cell tests. The CBE again performs the worst, displaying rapid capacity fade consistent with half‐cell cycling data (Figure 3c). These complementary measurements highlight that the main advantage of LHCEs lies in stabilizing the Sb/Gr electrode during alloying/dealloying rather than in mitigating dendritic growth on the counter potassium metal.

XPS analysis reveals pronounced differences in the interphase chemistry of Sb/C electrodes depending on the electrolyte. In the C1s region, electrodes cycled in CBE exhibit a dominant carbonyl (C═O) contribution, originating from carbonate solvent decomposition (EC:DEC). In contrast, the LHCE samples (G1, G2, and G1/G2) show a reduced C═O signal accompanied by stronger K2p features, indicating a lower extent of solvent‐derived surface species (Figure 3d). This trend is corroborated by the F1s and S2p spectra, where LHCE‐discharged electrodes display more intense KF signals together with sulfite/sulfate species arising from F─S bond cleavage and salt degradation, whereas CBE electrodes are comparatively enriched in organic components. The areal ratio comparison highlights this difference: CBE electrodes are dominated by C═O (∼80%) relative to KF (∼20%), while LHCEs exhibit a more balanced composition, with LHCE‐G1 approaching parity between inorganic and organic species (Figure S5). EDS mapping of potassiated Sb/Gr particles further supports the fluorine‐rich character of the interphase formed in LHCE‐G1: the LHCE‐G1 sample shows a homogeneous F distribution with 1.90 wt.% F (2.19 at.%), whereas no detectable F signal is observed for the particle discharged in EC:DEC; the sulfur content is likewise much higher in LHCE‐G2 (5.34 wt.%) than in EC:DEC (0.66 wt.%) (Figures S6, S7).

These findings correlate closely with the Raman measurements of the solvation shell, which demonstrated enhanced anion coordination in LHCEs and solvent‐dominated coordination in CBE. Taken together, the complementary spectroscopic evidence establishes a direct link between electrolyte solvation structure and the resulting interphase composition, providing a rationale for the superior electrochemical stability of LHCE‐based cells.

**FIGURE 4:**
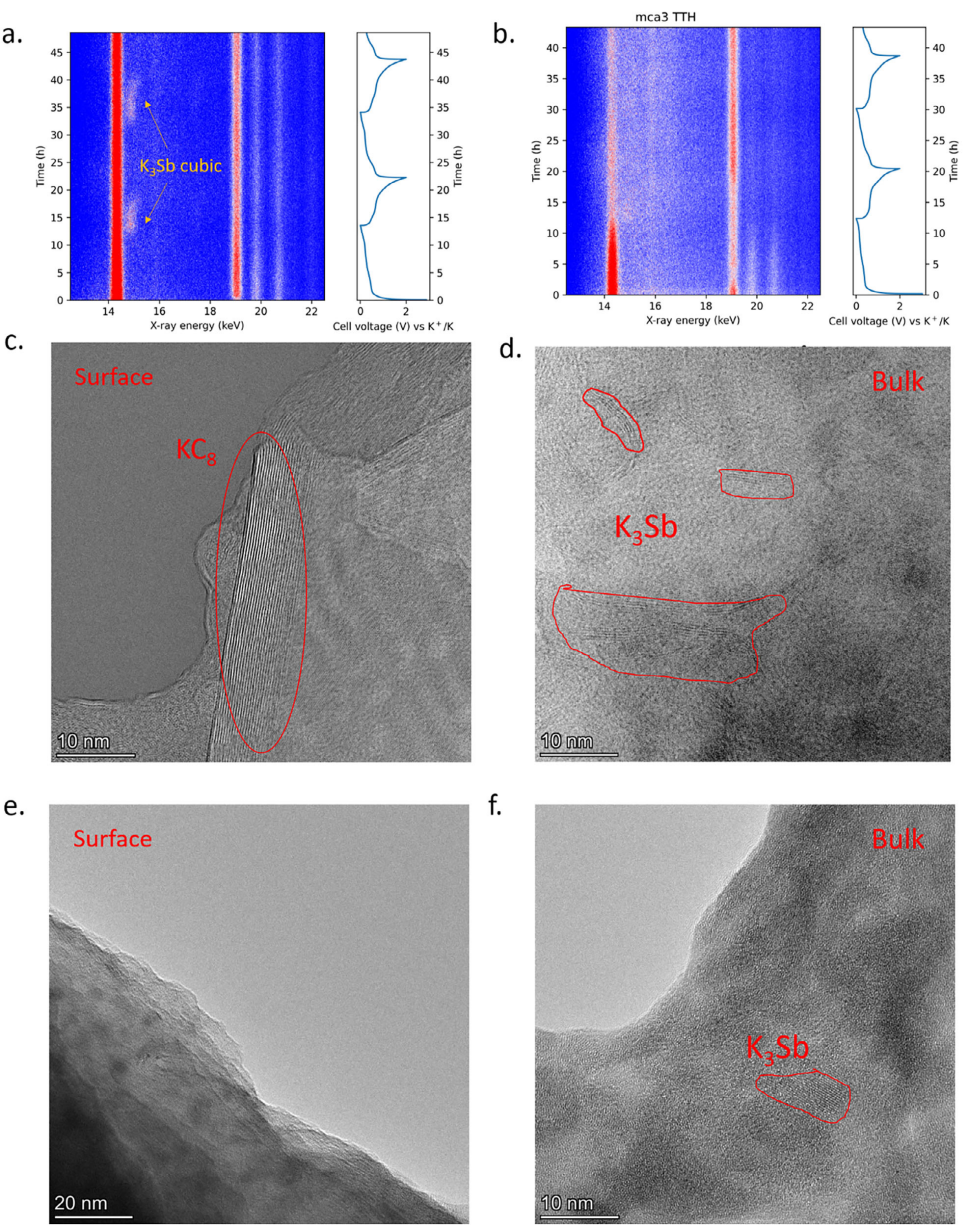
Structural analysis of the Sb/Gr electrode in two selected electrolytes. Operando ED‐XRD measurements reveal distinct phase evolution in (a) 1 M KFSI in EC:DEC electrolyte of 3 cycles and (b) LHCE‐G2 over two cycles. TEM images of the Sb/Gr electrode after the first discharge in (c,d) 1 M KFSI in EC:DEC and (e, f) LHCE‐G2 showing the morphology and crystallinity at the particle surface (c, e) and in the bulk (d, f). All measurements were done at 50 mA g^−1^ and at room temperature.

We subsequently carried out operando ED‐XRD and Raman spectroscopy to gain deeper insight into how the electrolyte composition governs the (de)potassiation pathway of the Sb/Gr electrode. These complementary techniques were selected because ED‐XRD can resolve crystalline phase evolution in real time, while Raman spectroscopy is sensitive to both crystalline and disordered environments, thus providing a more complete picture of alloying reactions that are known to proceed through amorphous intermediates. For clarity, we compared the best‐performing LHCE (LHCE‐G2) directly with the carbonate baseline reference (CE). Operando ED‐XRD experiments were conducted using a custom‐designed Huber diffractometer with a tungsten X‐ray tube providing a broad energy spectrum [31]. and a modified coin‐cell for diffraction measurements in transmission geometry (Figure 4a,b).

For the baseline electrolyte (Figure 4a), we observed the appearance of a small reflection at 14.8 keV at the end of discharge, which we assign to hkl = 220 of the cubic K_3_Sb phase. Further, the 311‐reflection of K_3_Sb is visible at 17.4 keV following the same behavior during cycling. These reflections vanished rapidly upon charging, consistent with a reversible two‐phase reaction between Sb and K_3_Sb. The absence of additional reflections indicates that intermediate phases: KSb_2_, KSb, K_5_Sb_4_ are largely amorphous [32], in agreement with previous operando XRD reports on K─Sb alloying mechanism [23, 33]. The reversible formation of K_3_Sb, however, implies significant lattice expansion and contraction, which is likely to impose mechanical stress on the composite electrode and accelerate degradation.

In sharp contrast, the ED‐XRD patterns showed less evidence of crystalline K_3_Sb formation for the LHCE‐G2 electrolyte. Instead, the dominant feature was a progressive decrease in the intensity of the pristine Sb peak at 14.2 keV, especially without the emergence of new crystalline reflections during the second charge‐discharge cycle. This observation suggests that in the LHCE‐G2 environment, the potassiation pathway is fundamentally altered; the reaction appears to terminate at amorphous or nanocrystalline K_x_Sb domains, avoiding the nucleation of bulk K_3_Sb (Figure 4b). Such behavior likely originates from kinetic constraints imposed by the altered solvation environment of LHCE G2, which suppresses deep alloying and the large volume fluctuations associated with the formation of crystalline K_3_Sb. This deviation in the reaction pathway provides a structural rationale for the markedly improved cycling stability observed with LHCE G2 compared with the CE. Transmission electron microscopy (TEM) of discharged electrodes in the CBE reveals distinct nanocrystalline domains of K_3_Sb within the Sb‐rich regions of the composite, together with partial crystallization of the carbon shell, suggesting potassium‐ion intercalation into the graphitic layers (Figure 4c,d). In contrast, TEM images of electrodes cycled in LHCE G2 show crystalline domains confined to the surface, while the particle cores remain largely amorphous with smaller and scarcer nanocrystalline domains (Figure 4e,f), consistent with the operando ED‐XRD observations. The coexistence of crystalline inclusions within an amorphous matrix in the CBE case inevitably generates internal mechanical stress during repeated potassiation and depotassiation, leading to particle cracking and accelerated capacity fade. In LHCE G2, the partial suppression of this multiphase crystallization, combined with the formation of a KF‐rich, mechanically resilient SEI, preserves the structural integrity of both Sb and carbon components and underpins the superior electrochemical stability of the composite electrode.

**FIGURE 5:**
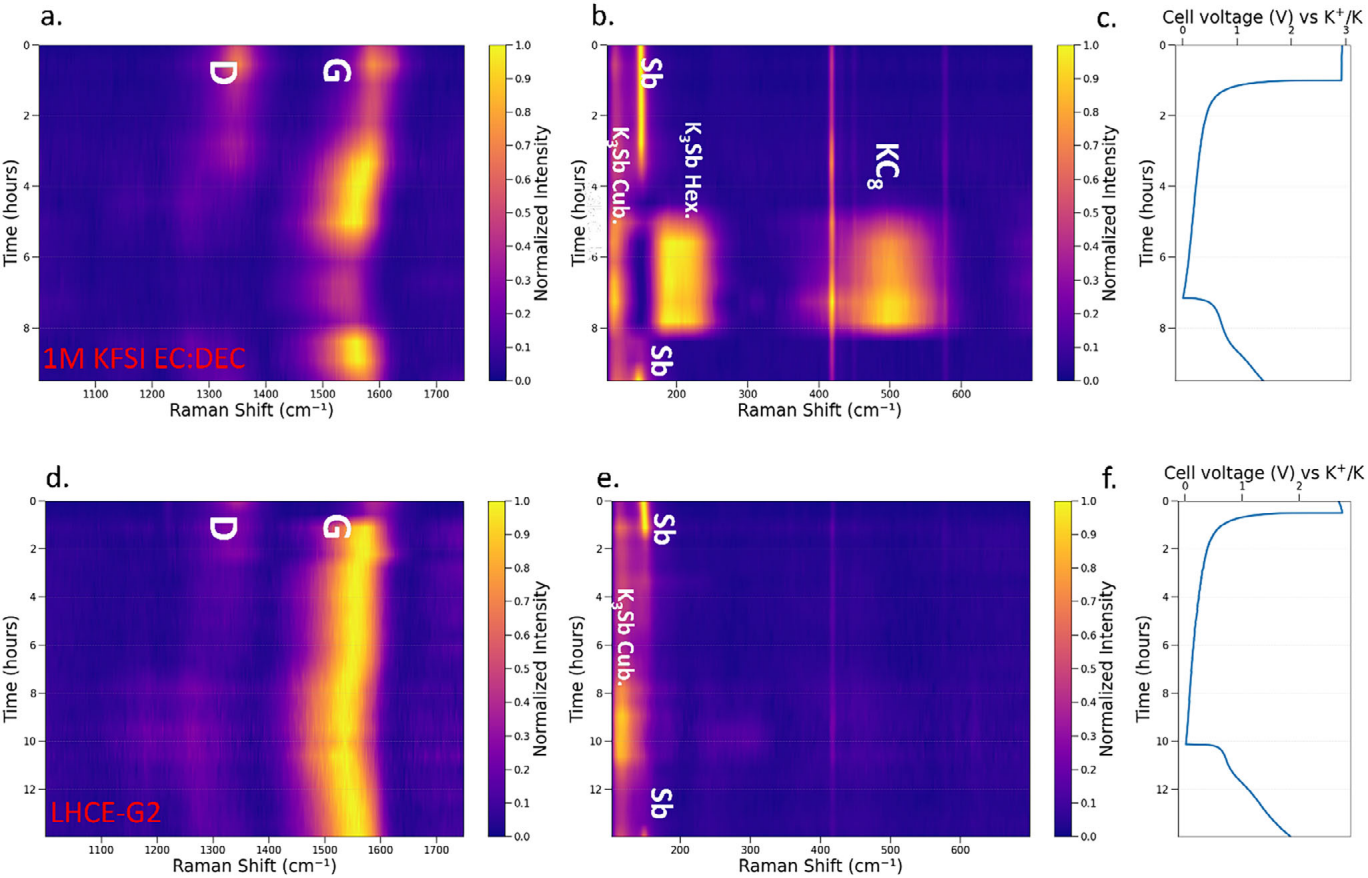
Operando Raman spectroscopy analysis of the Sb/Gr electrode in two selected electrolytes: (a–c) 1 M KFSI in EC:DEC (conventional electrolyte) and (d–f) LHCE‐G2. Both measurements were done at 25 mA g^−1^ current density at room temperature.

Since no graphite reflections were detected in the operando ED‐XRD patterns due to its lower weight proportion (30%) and its weak scattering nature, we employed operando Raman spectroscopy to follow the evolution of both the graphitic and alloying components of the Sb/Gr composite during cycling. The analysis first focused on the D and G bands, which correspond to disordered and graphitic carbon domains, respectively. In the pristine state, the intensity ratio I_D_/I_G_ ≈ 1.1, higher than that of the graphite precursor (I_D_/I_G_ = 0.7), indicates partial amorphization of the carbon shell induced by the high‐energy ball‐milling process (Figure S2). During the first discharge (Figure 5a,d), the G band gradually redshifts and becomes sharper and more intense at the beginning of discharge, consistent with potassium intercalation into the graphitic layers, as also observed in TEM. In literature, alkali‐ion intercalation in graphite typically causes an initial blueshift of the G band due to electron doping and occupation of π* antibonding states, followed by a redshift at later stages associated with tensile strain from lattice expansion [34, 35]. In our case, however, only the redshift is observed across all electrolytes, suggesting that the strain effect dominates the Raman response. This strain‐driven behavior likely arises from the highly disordered and nanoconfined nature of the graphitic domains and their mechanical coupling with the expanding Sb phase during potassiation.

At lower frequencies (Figure 5b,e), additional Raman bands provide insight into the concurrent alloying reactions occurring in the Sb component of the composite. In LHCE‐G2, the characteristic Sb vibration at 150 cm^−^^1^ diminishes rapidly at the onset of discharge, indicating early amorphization, while the band at 125 cm^−^^1^, attributed to cubic K_3_Sb, appears only near the end of discharge. In contrast, in the CBE, the Sb band remains intense up to roughly 50% state of discharge before decreasing and giving rise to two new features at 125 and 200 cm^−^^1^. The first corresponds to the cubic K_3_Sb phase, exhibiting a redshift relative to the bulk reference (145 cm^−1^) [9] due to phonon confinement from strain and nanoscale particle size [36, 37]. The second, at 200 cm^−^^1^, is assigned to the hexagonal K_3_Sb polymorph, consistent with previous reports for hexagonal Na_3_Sb [33, 38]. Alongside these alloying‐related modes, a new band develops near 500 cm^−^^1^ exclusively in the CBE (Figure 5e), which can be assigned to the intercalant–carbon lattice vibration characteristic of stage‐I KC_8_, marking deep potassium intercalation in the graphitic framework [39]. Notably, the emergence of both the hexagonal K_3_Sb and KC_8_ bands coincides with a sharp decrease in G‐band intensity, demonstrating that deep graphite potassiation occurs simultaneously with multiphase K–Sb alloy formation. During charge, the reverse transformations are observed in both electrolytes: The K_3_Sb bands disappear at the beginning of charge, the Sb band at 150 cm^−^^1^ reappears near the end of charge, and the G band blueshifts back to its original position at approximately 1580 cm^−^^1^.

The electrochemical response of bare graphite in both electrolytes further supports these spectroscopic observations. In the CBE, graphite delivers a reversible intercalation capacity characteristic of repeated potassium insertion and extraction, consistent with the appearance of the KC_8_‐related band at 500 cm^−^^1^ at the end of discharge (Figure S8a). In contrast, in LHCE‐G2, the first discharge exhibits only a small irreversible capacity followed by negligible electrochemical activity (Figure S8b), indicating that potassium insertion into graphite is strongly hindered in this electrolyte. The absence of the KC_8_ feature in the corresponding Raman spectra corroborates this finding and confirms that no low‐stage (K‐rich) intercalation compounds are formed. Consequently, the G‐band redshift observed in LHCE‐G2 cannot originate from charge‐transfer interactions associated with GIC staging but rather from mechanical strain transmitted from the expanding K─Sb alloy domains. The Raman response in LHCE‐G2 therefore, reflects elastic coupling between the carbon framework and the alloying phase, highlighting the mechanically constrained nature of potassiation in this electrolyte.

### 2.3 Operando Electrochemical Dilatometry (ECD)

**FIGURE 6:**
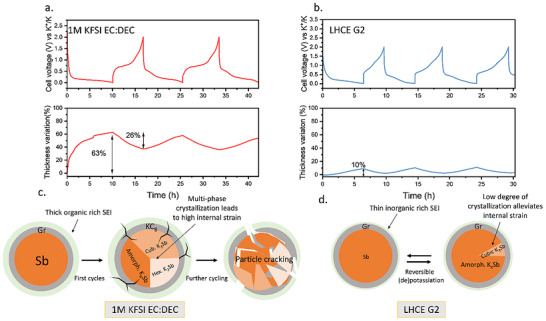
Operando dilatometry analysis of the Sb/Gr electrode cycled in the selected electrolytes: (a) 1 M KFSI in EC:DEC and (b) LHCE‐G2. Schematic representation of the (de)potassiation process and its mechanical repercussions in (c) the conventional electrolyte and (d) LHCE‐G2. Both measurements were performed at a current density of 50 mA g^−^^1^ at 25°C.

While operando Raman spectroscopy captures the chemical evolution of the active material, it does not directly reflect the accompanying mechanical stresses that develop within the electrode. To address this limitation, operando electrochemical dilatometry (ECD) was employed to quantify dimensional variations during cycling, thereby linking spectroscopic and mechanical responses. Figure 6 summarizes the results obtained for the composite Sb/Gr electrode cycled in the two selected electrolytes.

In the case of the CBE, the electrode exhibits a substantial thickness increase of up to 63% during the first potassiation, with a non‐linear slope indicative of potential‐dependent expansion associated with the multiphase K─Sb alloying process and the intercalation of potassium into the graphitic shell, as revealed by Raman spectroscopy. Upon depotassiation, the electrode fails to fully recover its original thickness, resulting in an irreversible expansion of ≈37%, which can be attributed to the incomplete reversibility of K–Sb phase formation, particle rearrangement, and SEI buildup. During subsequent cycles, the electrode undergoes smaller volumetric changes of around 26% (Figure 6a). For comparison, if a planar Sb film of 100 µm thickness and 10 mm diameter were to expand, in a single direction due to cell constraints, by 400%, as expected for bulk Sb during full potassiation, the resulting volume increase would correspond to a fivefold expansion, translating to a theoretical thickness of approximately 500 µm (a net change of ≈ 400 µm). This highlights that the experimentally observed 63% expansion in the Sb/Gr composite is drastically mitigated compared to the pure Sb case. Such a reduction can be attributed to the unique core–shell architecture produced by high‐energy ball milling, where the carbon shell buffers the volumetric changes of Sb and maintains the mechanical integrity of the electrode as opposed to already reported performances of bulk Sb anodes [40].

In contrast, when cycled in the LHCE‐G2 electrolyte, the electrode displays a markedly reduced swelling behavior, with the thickness increasing by only about 10% during the first discharge. Unlike the CBE case, the expansion–contraction profile shows nearly perfect reversibility, despite the lower specific capacity (Figure 6b). The thickness evolution is also more linear, suggesting a less abrupt alloying process. This observation supports the Raman and operando ED‐XRD findings, which point to the formation of a predominantly amorphous and reversible K_x_Sb phase with minimal participation of the graphitic shell. The reduced irreversibility further implies the formation of a thinner, KF‐rich interphase in LHCE‐G2, whereas a thicker and less compact SEI is formed in the CBE, as evidenced by XPS measurements. It is also worth noting that the smaller volume expansion observed in LHCE‐G2 may partially stem from the inherently lower capacity delivered by this electrolyte, both in the operando configuration and in coin‐cell tests. Nevertheless, the improved reversibility during the first cycle is independent of this capacity difference and reflects a more favorable (de)potassiation mechanism, which ultimately contributes to the enhanced structural and electrochemical stability.

The implications of these contrasting behaviors are schematized in Figure 6c,d. In the CBE, the large volumetric expansion arising from multiphase crystalline K–Sb alloy formation, together with the internal strain induced by concurrent (de)potassiation of the graphitic shell, promotes particle cracking and contributes to the rapid capacity fade (Figure 6c). In sharp contrast, the limited expansion, gradual alloying reaction, and formation of a thinner, inorganic‐rich SEI in the LHCE collectively provide a more mechanically stable framework, enabling prolonged cycling life (Figure 6d).

## 3 Conclusion

In summary, this study demonstrates that electrolyte formulation has a decisive impact on both the electrochemical and mechanical stability of Sb/Gr composite anodes in potassium‐ion batteries and does not merely help passivate the potassium metal surface in half‐cells. We demonstrate that an LHCE consisting of KFSI as a salt, diglyme as a solvent, and TTE as a diluent provides excellent cycling stability for Sb/Gr composite anodes reaching more than 300 cycles while keeping the maximum capacity obtained of 400 mAh g^−1^.

Using a variety of surface and bulk sensitive analytical methods, including operando Raman spectroscopy, ED‐XRD, electrochemical dilatometry, ex‐situ XPS and TEM, a direct correlation between the chemical, structural, and dimensional evolution of the electrode during cycling can be established. The conventional carbonate electrolyte promotes the formation of crystalline multiphase K–Sb alloys and a thick, organic‐rich SEI, which leads to large volume changes (>25%) and mechanical degradation, which in turn leads to inevitable capacity fade. In contrast, the LHCE‐G2 electrolyte favors reversible amorphous K_x_Sb formation, limits electrode swelling (∼10%), and produces a thinner, inorganic‐rich interphase, resulting in improved structural integrity and cycling stability. While the present results establish this behavior for Sb/Gr composite anodes, extension of the same mechanism to other alloy‐type anodes cannot be assumed because their phase evolution, reaction potentials, and volume changes differ substantially.

Beyond these findings, the takeaway message from our study is that electrolyte studies should focus on interfacial chemistry as well as bulk electrode processes. A comprehensive understanding of electrolyte–electrode interactions requires examining how interfacial reactions influence the bulk structure, mechanics, and long‐term electrochemical behavior of the electrode. We hope that this approach will encourage further studies aimed at uncovering the deeper electrochemical mechanisms that govern electrode performance and degradation.

## Author Contributions

**Ezzoubair Bendadesse**: conceptualization, investigation, writing – original draft, methodology, visualization, formal analysis, writing – review and editing, validation. **Max Wacha**: investigation, formal analysis. **Zeynep Erdöl**: investigation, formal analysis. **Yanan Sun**: formal analysis, investigation, writing – review and editing. **Carsten Prinz**: investigation, formal analysis. **Mirko Boin**: investigation, formal analysis, writing – review and editing. **Manuela Klaus**: investigation, formal analysis, writing – review and editing. **Tatiana Mishurova**: investigation, formal analysis, writing – review and editing. **Till Wolfram**: investigation, formal analysis. **Jürgen Janek**: writing – review and editing. **Philipp Adelhelm**: funding acquisition, writing – review and editing, visualization, validation, formal analysis, resources.

## Conflicts of Interest

The authors declare no conflicts of interest.

## Supporting information




**Supporting File**: advs76057‐sup‐0001‐SuppMat.docx.

## Data Availability

The data that support the findings of this study are available from the corresponding author upon reasonable request.

## References

[1] Y. Gao , Q. Yu , H. Yang , J. Zhang , and W. Wang , “The Enormous Potential of Sodium/Potassium‐Ion Batteries as the Mainstream Energy Storage Technology for Large‐Scale Commercial Applications,” Advanced Materials 36 (2024): 2405989, 10.1002/adma.202405989. 38943573

[2] R. Rajagopalan , Y. Tang , X. Ji , C. Jia , and H. Wang , “Advancements and Challenges in Potassium Ion Batteries: A Comprehensive Review,” Advanced Functional Materials 30 (2020): 1909486, 10.1002/adfm.201909486.

[3] Y. Xu , Y. Du , H. Chen , et al., “Recent Advances in Rational Design for High‐Performance Potassium‐Ion Batteries,” Chemical Society Reviews 53 (2024): 7202–7298, 10.1039/D3CS00601H. 38855863

[4] X. Min , J. Xiao , and M. Fang , “Potassium‐Ion Batteries: Outlook on Present and Future Technologies,” Energy & Environmental Science 14 (2021): 2186–2243, 10.1039/D0EE02917C.

[5] X. Du , Y. Gao , and B. Zhang , “Building Elastic Solid Electrolyte Interphases for Stabilizing Microsized Antimony Anodes in Potassium Ion Batteries,” Advanced Functional Materials 31 (2021): 2102562, 10.1002/adfm.202102562.

[6] Y. Domi , H. Usui , K. Kuritani , et al., “Anode Properties of Sb‐Based Alloy Electrodes for K‐Ion Batteries in an Ionic‐Liquid Electrolyte,” ACS Applied Energy Materials 6 (2023): 11583–11591, 10.1021/acsaem.3c02002.

[7] K. Song , C. Liu , L. Mi , S. Chou , W. Chen , and C. Shen , “Recent Progress on the Alloy‐Based Anode for Sodium‐Ion Batteries and Potassium‐Ion Batteries,” Small 17 (2021): 1903194. 10.1002/smll.201903194 31544320

[8] H. Gao , X. Guo , S. Wang , F. Zhang , H. Liu , and G. Wang , “Antimony‐Based Nanomaterials for High‐Performance Potassium‐Ion Batteries,” EcoMat 2 (2020): 12027.

[9] Z. Yi , N. Lin , W. Zhang , W. Wang , Y. Zhu , and Y. Qian , “Preparation of Sb Nanoparticles in Molten Salt and Their Potassium Storage Performance and Mechanism,” Nanoscale 10 (2018): 13236–13241, 10.1039/C8NR03829E. 29971315

[10] S. Choi , S. Kim , K. Yang , M. Cho , and Y. Lee , “Highly Stable Potassium‐Ion Battery Enabled by Nanoengineering of an Sb Anode,” ACS Applied Materials & Interfaces 14 (2022): 17175–17184, 10.1021/acsami.1c24251. 35389632

[11] Z. Wang , S. Qiao , M. Ma , et al., “High‐Entropy Conversion‐Alloying Anode Material for Advanced Potassium‐Ion Batteries,” ACS Nano 19 (2025): 15148–15160, 10.1021/acsnano.5c03792. 40214140

[12] Z. Yi , Y. Qian , J. Tian , K. Shen , N. Lin , and Y. Qian , “Self‐Templating Growth of Sb_2_Se_3_@C Microtube: A Convention‐Alloying‐Type Anode Material For Enhanced K‐Ion Batteries,” Journal of Materials Chemistry A 7 (2019): 12283–12291, 10.1039/C9TA02204J.

[13] J. Zheng , Y. Yang , X. Fan , et al., “Extremely Stable Antimony–Carbon Composite Anodes For Potassium‐Ion Batteries,” Energy & Environmental Science 12 (2019): 615–623, 10.1039/C8EE02836B.

[14] Q. Liu , L. Fan , R. Ma , et al., “Super Long‐Life Potassium‐Ion Batteries Based On An Antimony@Carbon Composite Anode,” Chemical Communications 54 (2018): 11773–11776, 10.1039/C8CC05257C. 30277235

[15] R. Zhang , H. Xue , T. A. Otitoju , et al., “Analogous Chelation to Boost Utilization of Sb in Sb Nanoparticles and N‐doped Carbon Composites for Enhancing Potassium Storage,” ACS Applied Materials & Interfaces 16 (2024): 40894–40902, 10.1021/acsami.4c06012. 39056581

[16] M. Ghosh , N. Yadav , and P. Adelhelm , “Glyme‐based Localized High Concentration Electrolytes Improve the Stability of Na‐ion Battery Materials in Half‐cells,” Batteries & Supercaps 8 (2025): 202400744.

[17] L. Qin , N. Xiao , J. Zheng , Y. Lei , D. Zhai , and Y. Wu , “Localized High‐Concentration Electrolytes Boost Potassium Storage in High‐Loading Graphite,” Advanced Energy Materials 9 (2019): 1902618, 10.1002/aenm.201902618.

[18] P. Nie , M. Liu , W. Qu , et al., “Unravelling the Solvation Structure and Interfacial Mechanism of Fluorinated Localized High Concentration Electrolytes in K‐ion Batteries,” Advanced Functional Materials 33 (2023): 2302235, 10.1002/adfm.202302235.

[19] X. Du and B. Zhang , “Robust Solid Electrolyte Interphases In Localized High Concentration Electrolytes Boosting Black Phosphorus Anode For Potassium‐Ion Batteries,” ACS Nano 15 (2021): 16851. 34633188 10.1021/acsnano.1c07414

[20] G. A. Ferrero , G. Åvall , K. Janßen , et al., “Solvent Co‐Intercalation Reactions for Batteries and Beyond,” Chemical Reviews 125 (2025): 3401–3439, 10.1021/acs.chemrev.4c00805. 40088123 PMC11951085

[21] W. Jiang , G. Nadeau , K. Zaghib , and K. Kinoshita , “Thermal Analysis of the Oxidation of Natural Graphite — Effect of Particle Size,” Thermochimica Acta 351 (2000): 85–93, 10.1016/S0040-6031(00)00416-0.

[22] R. G. Orman and D. Holland , “Thermal Phase Transitions in Antimony (III) Oxides,” Journal of Solid State Chemistry 180 (2007): 2587–2596, 10.1016/j.jssc.2007.07.004.

[23] C. Han , K. Han , X. Wang , et al., “Three‐Dimensional Carbon Network Confined Antimony Nanoparticle Anodes For High‐Capacity K‐Ion Batteries,” Nanoscale 10 (2018): 6820–6826, 10.1039/C8NR00237A. 29595204

[24] P. J. Carvalho , C. H. G. Fonseca , M.‐L. C. J. Moita , Â. F. S. Santos , and J. A. P. Coutinho , “Thermophysical Properties of Glycols and Glymes,” Journal of Chemical & Engineering Data 60 (2015): 3721–3737, 10.1021/acs.jced.5b00662.

[25] S. Tang and H. Zhao , “Glymes as Versatile Solvents For Chemical Reactions And Processes: From The Laboratory To Industry,” RSC Advances 4 (2014): 11251. 24729866 10.1039/C3RA47191H PMC3981120

[26] J. Wang , Y. Yamada , K. Sodeyama , C. H. Chiang , Y. Tateyama , and A. Yamada , “Superconcentrated Electrolytes for a High‐Voltage Lithium‐Ion Battery,” Nature Communications 7 (2016): 12032, 10.1038/ncomms12032. PMC4931331 27354162

[27] N. Xiao , W. D. McCulloch , and Y. Wu , “Reversible Dendrite‐Free Potassium Plating and Stripping Electrochemistry for Potassium Secondary Batteries,” Journal of the American Chemical Society 139 (2017): 9475–9478, 10.1021/jacs.7b04945. 28662577

[28] Y. Yamada , M. Yaegashi , T. Abe , and A. Yamada , “A Superconcentrated Ether Electrolyte for Fast‐Charging Li‐Ion Batteries,” Chemical Communications 49 (2013): 11194, 10.1039/c3cc46665e. 24150285

[29] P. N. Le Pham , V. Gabaudan , A. Boulaoued , et al., “Potassium‐Ion Batteries Using KFSI/DME Electrolytes: Implications of Cation Solvation on the K^+^‐Graphite (Co‐) Intercalation Mechanism,” Energy Storage Materials 45 (2022): 291–300.

[30] M. Exner , D. Stepien , A. I. Freytag , et al., “Electrolyte‐Dependent Sodium Plating for Anode‐Free Na‐Ion Batteries Studied by Operando Optical Microscopy,” Advanced Science 13 (2026): 00058. 10.1002/advs.202600058 PMC13115960 41717857

[31] D. Apel , M. Meixner , A. Liehr , et al., “Residual Stress Analysis Of Energy‐Dispersive Diffraction Data Using A Two‐Detector Setup: Part II — Experimental Implementation,” Nuclear Instruments and Methods in Physics Research Section A: Accelerators, Spectrometers, Detectors and Associated Equipment 877 (2018): 56–64, 10.1016/j.nima.2017.09.006.

[32] J. Sangster and A. D. Pelton , “The K‐Sb (Potassium‐Antimony) System,” Journal of Phase Equilibria 14 (1993): 510–514, 10.1007/BF02671972.

[33] V. Gabaudan , R. Berthelot , L. Stievano , and L. Monconduit , “Inside the Alloy Mechanism of Sb and Bi Electrodes for K‐Ion Batteries,” Journal of Physical Chemistry C 122 (2018): 18266–18273, 10.1021/acs.jpcc.8b04575.

[34] J. Zou , C. Sole , N. E. Drewett , M. Velický , and L. J. Hardwick , “In Situ Study of Li Intercalation Into Highly Crystalline Graphitic Flakes of Varying Thicknesses,” The Journal of Physical Chemistry Letters 7 (2016): 4291–4296, 10.1021/acs.jpclett.6b01886. 27740774

[35] J. C. Chacón‐Torres , L. Wirtz , and T. Pichler , “Manifestation of Charged and Strained Graphene Layers in the Raman Response of Graphite Intercalation Compounds,” ACS Nano 7 (2013): 9249–9259, 10.1021/nn403885k. 24025089 PMC3807528

[36] A. K. Arora , M. Rajalakshmi , T. R. Ravindran , and V. Sivasubramanian , “Raman Spectroscopy of Optical Phonon Confinement In Nanostructured Materials,” Journal of Raman Spectroscopy 38 (2007): 604–617, 10.1002/jrs.1684.

[37] E. S. F. Neto , S. W. da Silva , P. C. Morais , M. I. Vasilevskiy , M. A. Pereira‐da‐Silva , and N. O. Dantas , “Resonant Raman Scattering in CdS_x_Se_1−x_ Nanocrystals: Effects of Phonon Confinement, Composition, and Elastic Strain,” Journal of Raman Spectroscopy 42 (2011): 1660–1669, 10.1002/jrs.2918.

[38] M. B. Tzolov and M. N. Iliev , “Raman Scattering From Monoalkali (Na‐Sb and K‐Sb), Bialkali (Na‐K‐Sb) and Multialkali (Na‐K‐Sb‐Cs) Photocathodes,” Thin Solid Films 213 (1992): 99–102, 10.1016/0040-6090(92)90481-P.

[39] M. S. Dresselhaus and G. Dresselhaus , “Intercalation Compounds of Graphite,” Advances in Physics 51 (2002): 1–186, 10.1080/00018730110113644.

[40] D. Liu , L. Yang , Z. Chen , et al., “Ultra‐Stable Sb Confined Into N‐doped Carbon Fibers Anodes For High‐Performance Potassium‐Ion Batteries,” Science Bulletin 65 (2020): 1003–1012, 10.1016/j.scib.2020.03.019. 36659015

