# Supplementary material for: Impact of Electrolyte Formulation on the Phase Behavior and Interphase Formation of Sb/Graphite Electrodes for K‐Ion Batteries
Source: Adv Sci (Weinh). 2026 Jun 11;13(50):e76057. doi: 10.1002/advs.76057 (PMC13337059; doi:10.1002/advs.76057)
Supplement: Supplementary file 1 [file ADVS-13-e76057-s001.docx]

**Supporting information on**

**Impact of electrolyte formulation on the phase behavior and interphase formation of Sb/Graphite electrodes for K-ion batteries**

*Ezzoubair Bendadesse^a^*, Max Wacha^b^, Zeynep Erdöl^a^*, *Yanan Sun^a,d^, Carsten Prinz^c^, Mirko Boin^e^, Manuela Klaus^e^, Tatiana Mishurova^e^, Till Wolfram^f^, Jürgen Janek^b^ Philipp Adelhelm^a,d^*.*

*^a^ Institut für Chemie, Humboldt Universität zu Berlin, Brook-Taylor Str. 2, 12489 Berlin, Germany.*

*^b^* *Justus-Liebig-Universität Gießen, Heinrich-Buff-Ring 17, 35392 Gießen, Germany.*

*^c^ BAM Federal Institute for Materials Research and Testing, Richard-Willstätter-Str. 11, 12489 Berlin, Germany.*

*^d^Joint Research group for operando battery analysis (CE-GOBA), Helmholtz Zentrum Berlin für Materialien und Energie (HZB), Hahn-Meitner-Platz 1, 14109 Berlin, Germany*

*^e^Dept. Microstructure & Residual Stress Analysis (CE-AME), Helmholtz Zentrum Berlin für Materialien und Energie (HZB), Hahn-Meitner-Platz 1, 14109 Berlin, Germany.*

*^f^Wolfram Chemie GmbH, Johann-Hittorf-Straße 8, 12489 Berlin, Germany.*

Keywords: Potassium-ion batteries, Operando analysis, Alloy anodes, Sb-Graphite.

*** Corresponding authors:** Ezzoubair.bendadesse@hu-berlin.de,

Philipp.Adelhelm@hu-berlin.de

**Experimental section**

***Synthesis of Sb/Gr***: Commercial powders of Sb (Alfa Aesar, -100 mesh, 99.5%) and Graphite (Wolfram Chemie, 99.9%) were mixed using high energy ball milling under Ar atmosphere for 10h in a 7:3 (Sb:C) mass ratio. 10mm diameter Zirconia balls were used in a 10:1 ball-to-powder mass ratio. The resulting Sb/Gr powder was used as it is without further modification.

***Electrochemical measurements:*** Sb/Gr electrodes were made by mixing the active material with super P carbon additive and a blend of CMC and SBR polymers (8:2) as a binder in an 8:1:1 mass ratio using deionized water as a solvent for the binder. The slurry was then cast on carbon coated aluminum foil and 10mm diameter electrodes were punched out after drying the film with a loading of 1-2 mg cm^-2^. The electrodes were then transferred to Ar-atmosphere glove box (0.1 ppm H_2_O and O_2_) for coin-cell assembly. To evaluate the K-ion storage performances of the samples, CR2032 coin-type cells were assembled using Sb/Gr as the working electrode, K-metal as the counter electrode, GF/D glass fiber as the separator and the selected electrolyte of which the composition is listed below:

*Table S1. Molar composition of the LHCEs.*

| LHCE G1 | KFSI:DME:TTE @ 1:2:1 Molar ratio |
| --- | --- |
| LHCE G2 | KFSI:DGME:TTE @ 1:2:1 Molar ratio |
| LHCE G1/G2 | KFSI:DME:DGME:TTE @ 1:1:1:1 Molar ratio |

A widely used carbonate based electrolyte (CBE) was chosen as a reference point for performance comparison and consisted of 1M KFSI in EC:DEC (1:1 %vol).	The cells were cycled using a galvanostatic charge discharge protocol between 0.01-2 V vs K^+^/K using a BCS potentiostat (Biologic, France). 5 formation cycles were performed at 50 mA g^-1^ to ensure SEI formation and electrode passivation, afterwards the cells were cycled at 200 mA g^-1^. Symmetric cells were assembled in a similar manner only using the same material as both the counter and working electrodes. For Sb/Gr|Sb/Gr symmetric cells, the electrodes were pre-cycled in half cells to ensure SEI formation and contrasting SOCs, meanwhile K|K cells were assembled without any pre-treatment.

***Raman spectroscopy***: The solvation structure of each electrolyte and the (de)potassiation mechanism of the Sb/Gr electrodes were investigated using an inVia™ confocal Raman microscope (Renishaw, UK) equipped with a 532 nm green laser. Operando measurements were carried out in a commercial optical cell (EL-Cell, Germany) featuring a sapphire window. A strip of Sb/Gr composite served as the working electrode and was continuously probed during charge and discharge against a potassium metal counter electrode, while 25 µL of electrolyte were used to wet the GF/D separator. The Raman spectra were acquired in real-time during GCD of the electrochemical cell. 3 accumulations of each spectrum were acquired.

***X-ray photoelectron spectroscopy (XPS):*** Sb/Gr discharged in each of the selected electrolytes were collected from coin-cells inside an Ar-filled glove box to avoid contamination. The electrodes were discharged at 50 mA g^-1^ down to 0.01 V vs K^+^/K with an extra CC-CV step to ensure full SEI formation. For the XPS measurements of the four different types of samples a PHI VersaProbe IV system (Physical Electronics Inc.) was used equipped with a monochromated Al Kα X-ray source (X-ray source 50 W, 15 kV, beam diameter 200 μm). The samples were mounted on a sample holder using non-conductive double sided adhesive tape. This was done in a glovebox (M. Braun Inertgas-Systeme GmbH, O2 < 5 ppm, H2O < 5 ppm) filled with Argon. Transfer of the samples under inert conditions from the glovebox to the analysis chamber was performed using an air-tight transfer shuttle. For detailed spectra a step time of 20 ms, a step size of 0.2 eV and an analyzer pass energy of 55.00 eV were used. During measurements, the sample surface was charge-neutralized and the pressure ranged from 10−7 to 10−6 Pa. Data analysis and area ratio calculation was performed using the CasaXPS software (Version 2.3.26PR1.0). A calibration of the spectra was done using the Graphite Signal (C=C) C1s (284.5 eV), since the samples were composed of graphite. All components (besides C=C) were fitted with a Gaussian Lorentzian product formula (GL(30), 30% Lorentzian and 70% Gaussian). The graphite C=C signal was fitted with an asymmetric Lorentzian line shape (LA(1.2,2.5,5)) according to literature. Assignment of the components was also made according to literature.

***Operando Energy Dispersive X-Ray Diffraction (ED-XRD):*** modified coin-cells featuring a 8mm diameter mylar window were used to capture the evolution of the crystal structure of our samples in the selected electrolytes. A custom-designed 8-circle Huber diffractometer was used for ED-XRD operando measurements. The device is equipped with a conventional laboratory tungsten long fine focus X-ray tube generating a continuous spectrum of energies up to 60 keV. The coin-cells have been mounted on the diffractometer stage inside a circuit board connected to a potentiostat (see ECD below). The primary beam was defined by a polycapillary semi-lens and a slit with a diameter of 2 mm to aim at the center of the coin-cell window. The measurements were performed in transmission geometry. The diffraction signal was captured by a KETEK AXAS-M energy-dispersive silicon drift detector (SDD). The detector stage was set to a fixed diffraction angle of 2θ = 16°. In addition, a secondary 0.15° collimating soller optics was used in front of the detector. Continous counting of the diffraction signals was used during the charge and discharge of the coin-cells for multiple cycles.

***Operando Electrochemical Dilatometry (ECD):*** ECD measurements were performed using an ECD-nano cell device (EL-CELL GmbH, Germany) configured in a three-electrode setup. The working and counter electrodes were separated by a fixed glass-ceramic separator, ensuring that only the thickness change of the working electrode was recorded. Approximately 250 μL of electrolyte were added per cell. Prior to cycling, the assembled cells were allowed to rest for 12 h to ensure equilibration. ECD experiments were carried out on a Biologic SP-50 potentiostat at 25 °C, with GCD cycling performed between 0.01 and 2 V vs. K⁺/K. Electrode thickness variations were recorded simultaneously during the GCD measurements. The relative thickness evolution (%) shown in **Figure 6** was calculated using the following method:

$${\Delta h}_{rel}\text{=}\frac{h_{0}\text{-}h_{x}}{h_{0}}\times100$$

Where *h_x_* is the recorded thickness of the electrode during cycling and *h_0_* is the initial thickness of the electrode before cycling and of which we subtracted the aluminum foil thickness.

**Figure S1**. Thermogravimetric analysis of the Sb/Gr powder under O_2_ atmosphere. The measurement was perfromed at a 5°C/min heating rate.

**Figure S2.** Raman spectra of Sb/Gr electrode and pristine commercial graphite powder.

**Figure S3.** a) 1^st^ cycle (charge/discharge) of the Sb/Gr electrode cycled in the selected electrolytes. b) Cyclic Voltammetry profiles of the Sb/Gr electrode cycled in the selected electrolytes, the 5^th^ cycle is shown here.

**Figure S4.** Symmetric K|K cells in three LHCE electrolytes : a) LHCE G1, b) LHCE G2 and c) LHCE G1/G2.

**Figure S5.** Carbonate and KF XPS Peaks area comparison.

**Figure S6.** a) HAADF-STEM image and b-f) corresponding EDX elemental maps of a discharged Sb/Gr particle cycled in 1 M KFSI in EC:DEC. The distributions of Sb, C, S, O, and K are shown for the selected area.

*Table S2. Semi-quantitative EDX elemental composition of the selected Sb/Gr particle area after discharge in 1 M KFSI in EC:DEC, obtained from the STEM-EDX mapping shown in Figure S4. Atomic and mass fractions are reported for the detected elements.*

| Element | Atomic Fraction (%) | Weight Fraction (%) |
| --- | --- | --- |
| Sb | 4.03 | 21 |
| C | 44.91 | 23.99 |
| S | 0.47 | 0.66 |
| O | 33.56 | 23.88 |
| K | 17.03 | 29.62 |

**Figure S7.** a) HAADF-STEM image and b-f) corresponding EDX elemental maps of a discharged Sb/Gr particle cycled in LHCE-G1. The distributions of Sb, F, S, O, and K are shown for the selected area.

*Table S3. Semi-quantitative EDX elemental composition of the selected Sb/Gr particle area after discharge in LHCE-G1, obtained from the STEM-EDX mapping shown in Figure Sx. Atomic and mass fractions are reported for the detected elements.*

| Element | Atomic Fraction (%) | Weight Fraction (%) |
| --- | --- | --- |
| Sb | 5.46 | 26.41 |
| F | 2.77 | 2.09 |
| S | 1.48 | 1.88 |
| O | 38.57 | 24.5 |
| K | 19.03 | 29.54 |

**Figure S8.** Charge discharge voltage profile of the commercial graphite used in the Sb/Gr composite cycling in LHCE G2 (a) and 1M KFSI EC:DEC (b). 100 cycles are shown here.
